# An opinion: social workers serve vulnerable populations in various digital ways under the crisis of the COVID-19 pandemic

**DOI:** 10.3389/fpsyt.2023.1247769

**Published:** 2023-09-14

**Authors:** Li-Jie Du

**Affiliations:** Department of Sociology, Philosophy and Law and Politics College, Shanghai Normal University, Shanghai, China

**Keywords:** digital service, mental health, technology, wellbeing, social work, COVID-19, crisis, health communication

## 1. Introduction

Everyone's life has been impacted by the COVID-19 (coronavirus) pandemic, either directly through exposure to the virus or indirectly by measures taken to mitigate its spread. A social worker is a professional who works to improve the overall wellbeing of the general population, with a priority on the vulnerable, oppressed, and the poor, assisting in meeting the basic and complex needs of communities or individuals (1). The pandemic has highlighted the possibilities, benefits and challenges in the digital services provided by social workers on a global scale.

COVID-19 challenged the limitations of social workers' abilities in various fields as they provided essential services within uncertain and high-risk work conditions. In the early stages of the pandemic, the lack of PPE (Personal Protective Equipment) for those working on the front lines was one of the significant problems in some countries. Social workers may have come into contact with people carrying the coronavirus or contaminated surfaces/objects while assisting health professionals or visiting clients. Some social workers and social care workers have contracted the virus, or even died, from the COVID-19 pandemic. Those who benefited from their care have expressed their grief in various ways, although traditional media rarely reported their stories (2). In response to the pandemic, they were expected to comply with government prohibitions, subsequent safety measures, and maintain social distancing during the lockdown. They often worked remotely from home and used online services to protect children, older adults, and others in “at-risk” groups within difficult conditions mediated by digital technology. They were required to adopt videoconferencing or phone calls, to ensure continued engagement with service users where face-to-face contact was restricted (3).

This review article highlights services social workers provided to under-served populations in society by various digital ways, not limited to children and older adults, but also the poor, disabled, homeless, and others. Digital technologies bring new trends to current and future social work intervention, research and training. Due to space and word limitations of the “opinion type” article, this short review focuses mainly on two groups as examples: children and older adults.

## 2. Providing digital service for vulnerable groups: children and older adults

### 2.1. Continuous services and protective measures for children

Potential damage to children has been identified as a result of the COVID-19 pandemic, which disrupted the daily lives of millions of children and their families worldwide. Child protection works are achieved by social workers getting close to children, especially on home visits by immersing themselves in the routine life of the family, assisting the parents to change their attitudes and behaviors toward their children (4). Usually, home visiting involves close contact with the physical, sensory, and emotional world hidden in the private space of the family when implementing child protection (5). The COVID-19 crisis disrupted these taken-for-granted practices and presented the social workers with unique challenges (6). Although the pandemic affected the ability of Child Protection Services (CPS) to respond to children in need, the workers found ways to reach children at risk, for example, by establishing crisis hotlines and crisis houses (7). During the crisis, one-way that social workers avoided the risk of contracting and transmitting the virus was by undertaking the work they would previously have done in children's home, from their own homes using digital communication technologies (8). There is considerable research showing innovation in how social workers delivered services through phone and video calls from their homes. Communication technologies, such as smartphones, were used to keep in touch, to experience the sense of “being together” when physically separated, and to become part of the “mundane intimacy of everyday life” (9).

The limitations of digital connection for children are well known such as the lack of hardware, software and connectivity, as well as the usual concerns about possible miscommunication, misinterpretation and malicious use, etc. Social workers working from home rather than in the office were cut off from vital sources of peer and supervisory support (10). Although the use of digital media and technologies in home visiting has many limitations, it encourages social workers to explore how digital intimacy emerges in virtual home visiting. The findings suggest that social workers need to engage with their clients inner worlds, such as children's feelings about not being able to see their parents face-to-face, and their anxieties about doing things differently, as well as their outer worlds, including practical issues such as access to the internet and the functionality of video calls. Therefore digital communication is most effective when both the inner and outer worlds of those involved are considered (11). The new digital social work practice provides a meaningful and helpful model of communication for some children, parents, and other family members, as children sometimes reveal more about their lives and narrate their experience in a different and more fulsome way when communication is mediated through a screen, enabling greater intimate engagement compared to what social workers had achieved on in-person visits to the home (8).

After COVID-19, social work could once again be connected through in-person intimacy and digital intimacy, which must be understood as a hybrid practice that integrates digital practices such as video calls and face-to-face interactions (12), at least, for some families and children in need. Advances in digital technology made hybrid models to be implemented as quickly as possible, which online and offline services were both presented in the above case of home visits for children's protection and other fields.

### 2.2. Serving older adults in the community and in nursing facilities

The COVID-19 pandemic has been challenging for people of all ages, but particularly devastating to adults 65 and older. When local governments took the blockade actions to mitigate the spread of COVID-19, it had a double impact on older adults. It serves to shield them from the virus, with adverse side effects, including increased social isolation, delayed medical treatment, difficulty in meeting basic needs and ageism (13). Older adults died in disproportionately higher numbers, especially in long-term care facilities. There were some people displayed relief when they initially learned the majority of those dying from the virus were older adults. It reflected the ageism that seems to exist in our society (14). The social work profession plays huge roles in advocating for social inclusion, supporting older people to find ways to overcome social isolation, receive information, and access resources, including increased usage of assistive technology.

The older adults were encouraged to stay at home and received more strict instructions on social distancing (15). Social isolation and loneliness have been linked to adverse physical and mental health outcomes, including increased depression and anxiety symptoms (16). Social workers and other gerontologists are increasingly voicing concerns about loneliness and social isolation among older adults. Social workers increased advance care planning, to reduce social isolation, and expand the use of on-line technology for service provision (17).

Maintaining physical distancing has limited social workers' interaction and care for the older adults. Social workers explored promoting kinship care and support practices as an alternative mechanism to meeting the welfare of older people (18). Because residents and staff in long-term care (LTC) facilities were at high risk for infection during the COVID-19 pandemic, supervision of LTC facilities was a crucial issue (19). Social workers assisted residents in maintaining relationships with their families as visiting restrictions were implemented in many nursing homes. Examples of practices by social workers redeployed to nursing homes include facilitating residents to see and talk to family members via social media apps, asking families to purchase tablets for the residents to allow them to maintain regular contact (20).

## 3. Discussion

### 3.1. Innovation and development of digital services under crisis

Technology presented opportunities for the continuation of social services during the global pandemic. There were no existing models for transferring social workers online practices in a working-from-home context during COVID-19. Some social workers adapted by conducting phone and video calls from their own homes, pioneering efficient digital services and online visits among other things (8). Asynchronous telecommunication, such as the use of the text messaging and emails, has also increased in practice as an adjunct to face-to-face engagement (21). There were some cases of co-produced qualitative research study on the experiences of social workers and disabled users, using digital technologies when communicating with each other in the service (22). With the increasing number of patients who had died from COVID-19 infections worldwide, social workers helped to tackle the issues highlighted by infections and deaths, which included dealing with grief and loss when regular routines were disrupted, and providing psychosocial support during and after the end of life, in various digital ways. Instead of following customary procedures when a resident passes away, the social workers had to coordinate with the resident's family and the funeral directors to meet the unique requirements of the pandemic management system (23), all while avoiding face-to-face contact at all times. There is still a need for further support for social workers to access flexible, efficient, and creative tools to maintain the quality of service delivery in the post-pandemic environment (24).

The emerging literature review on social work has revealed several benefits of using digital tools and online platforms, including providing services to the larger population and making social work more available and accessible (24). Some online training programs were implemented widely on digital social media platforms like Zoom during the pandemic. These platforms allowed the professional social work community to plan activities, get peer support or advice (23), and facilitate exchanges and cooperation. Social workers should serve vulnerable populations in their own communities, regions and countries, as well as work internationally to combat global public health crisis's (25). As an international organization, the International Association of Schools of Social Work(IASSW)disseminated digital publications through their website, presenting the response of social workers in different communities and organizations, sharing images to illustrate stories and experiences with a collection of country reports responding to the crisis and a particular focus on social work actions in supporting and caring for the most vulnerable populations (26).

### 3.2. Resistance from some social workers and the challenge associated with digital service delivery

Prior to the global COVID-19 pandemic, telephone and video conferencing had been used in the delivery of counseling services or therapeutic interventions for decades, but few studies did a rigorous analysis of both face-to-face and tele-mental health services for reliable comparisons, and understanding the impact on outcomes remains limited (27). Emerging literature reported the resistance to the exclusive use of technology from front line social workers practicing during the pandemic. When face-to-face contact was stopped, social workers who had well-established careers challenged the one-size-fits-all approach and were clear about the need to continue in-person meetings, using all necessary safety precautions, for meaningful engagement and accurate assessments (3). One experienced social worker shares the story of face-to-face contact with the older adults by offering “walking appointments”: they were not meeting in the older adults' homes but outdoors, such as going for a walk in the park together, which encourages physical activity and reduces social isolation, maintaining a safe social distance and allowing the social worker to continue in-person meetings (23).

The spread of the pandemic and the use of technology changed social work models, also challenged the capacity of social workers to respond to the change. They have been searching creative new ways to cope with all kinds of problems arising since the sudden outbreak of the pandemic. Some surveys and studies, exploring the impact of remote delivery and technology on relationship-based practice, reported how remote working arrangements were threatening the role of the team, which had implications for staff wellbeing and practice. This was particularly problematic for newly qualified social workers with no collegial relationships prior to the pandemic (28). The findings highlight negative outcomes on relationship building and social worker self-care, alongside concerns that efficiency would be prioritized over future face-to-face contact with service users (3). In the UK, the social work and social care sectors report the highest amount of mental health sickness among all professions (29). The prolonged poor and complicated working conditions affect the physical and mental health of the staff, so protecting and promoting their mental health should be prioritized, particularly when caused by alternative models of remote delivery without proper planning or staff training.

### 3.3. Digital technologies brought new trends to current and future social work training

The consequences of the COVID-19 pandemic brought new perspectives not only to social work interventions and the research, but also the training (30). The models of technology in social service delivery have been praised for increasing accessibility and reducing costs for service providers and users, especially in rural or under-resourced regions before COVID-19 (31). Strength from the online program is dependent on individuals' skills and the functionality of platforms being used. In practical terms, issues have emerged regarding the skills needed to engage with technology for service providers and users alike, which needed for online communication are not necessarily the same interpersonal skills developed in traditional social work training. Reflecting on the provision of online social work training during the pandemic, some reports highlight how camera angles influence how we use and maintain eye contact with interviewees, and how the way we communicate face-to-screen is profoundly different to face-to-face communication (32). It is a different challenge for educators and students in this new and unexpected environment, as social work has a practice component that involves field training particularly.

Digital communication technologies have fundamentally changed the provision of social work education, which was particularly pronounced in placement training. Social work students in many countries around the world are required to complete a certain number of credits of practice in order to get a diploma and professional license. Social work field education departments, industry partner agencies and accrediting bodies have needed to rapidly adapt how placements are conceptualize, and continued to provide student placements throughout the pandemic (33). Technological skills had brought new trends to social work education during the COVID-19 pandemic and will influence staff training programs in the future.

## 4. Conclusion

In many countries, social work has been recognized as an essential service. Social workers guided by values and ethics of their profession, are resisting the ailments in society and focusing their efforts on protecting the vulnerable groups (34). Under the crisis of COVID-19 pandemic, the delivery of face-to-face service had been reduced and replaced by various digital ways to communicate efficiently, which is still vital to maintain the professional relationship with the service users. It needs to be considered how these technological skills can be developed within mainstream social work education and integrated into the continued professional development, to prepare better for social workers in remote or online service delivery (3). The interdisciplinary collaboration between social work bodies and organizations with technology developers will improve technology-mediated social work practice to be aligned with professional principles, ethics, and values (24). Digital approaches hold potential for overcoming many challenges, yet barriers persist in ensuring access to quality, affordable, and effective services, so having a team of ethicists, clinicians, public, and mental health experts, individuals with lived experience, as well as computers scientists will be needed for successful implementation (35).

## Author contributions

The author confirms being the sole contributor of this work and has approved it for publication.
